# Endobronchial cryptococcosis without pulmonary parenchymal involvement caused by *Cryptococcus neoformans* in an immunocompetent host

**DOI:** 10.1002/rcr2.1343

**Published:** 2024-04-03

**Authors:** Yui Shimanuki, Susumu Sakamoto, Hiromichi Yoshida, Noboru Iizuka, Shion Miyoshi, Satoshi Sonobe, Naobumi Tochigi, Kazuma Kishi

**Affiliations:** ^1^ Department of Respiratory Medicine Toho University Omori Medical Center Tokyo Japan; ^2^ Department of Surgical Pathology Toho University Omori Medical Center Tokyo Japan

**Keywords:** bronchoscopy, *Cryptococcus neoformans*, endobronchial cryptococcosis

## Abstract

*Cryptococcus* is a yeast‐like fungus. Pulmonary lesions caused by *Cryptococcus neoformans* typically present as single or multiple nodules or infiltrative lesions in the lungs; however, endobronchial lesions are rare. A 40‐year‐old previously healthy Japanese man was referred to our hospital due to an abnormality detected on chest computed tomography. The analysis revealed focal bronchiectasis and bronchial wall thickening in the right upper lobe, which persisted for 6 months. Bronchoscopy showed reddish and edematous mucosa, stenosed bronchi (right B1 and B3), and white moss at the bifurcation of the right upper bronchus. Transbronchial biopsy revealed numerous yeast‐like fungi and an encapsulated body. Bronchial washing for fungus culture identified *Cryptococcus neoformans.* Although analysis for serum cryptococcal antigen was negative, bronchoscopy led to a definitive diagnosis. Antifungal treatment improved the bronchial wall thickening. This is a rare case of endobronchial cryptococcosis caused by *Cryptococcus neoformans* without pulmonary parenchymal involvement in an immunocompetent host.

## Graphical abstract

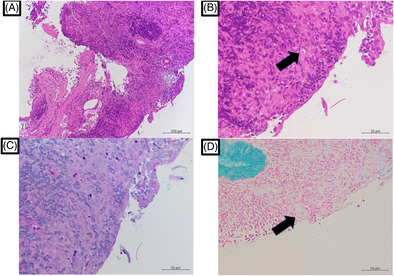

## INTRODUCTION

*Cryptococcus* is a type of yeast‐like fungus belonging to the Basidiomycetes family that exists in the environment. Two species cause infections in humans: *Cryptococcus neoformans* and *Cryptococcus gattii*. *C. neoformans* is often found in soil contaminated with bird droppings. Pulmonary cryptococcosis occurs both in healthy individuals and in immunocompromised patients. In general, pulmonary cryptococcosis is typically identified as a single well‐defined nodule or multiple nodules or consolidation, while endobronchial lesions are rare.^1^ In this article, we report a case of endobronchial cryptococcosis caused by *C. neoformans* without pulmonary parenchymal involvement in an immunocompetent host.

## CASE REPORT

A 40‐year‐old previously healthy Japanese male visited our hospital due to the detection of an abnormality on chest computed tomography (CT) performed during infection with coronavirus disease‐2019 (COVID‐19) 1 month earlier. Chest CT did not show pneumonia due to COVID‐19. However, localized bronchiectasis and bronchial wall thickening were detected in the right upper lobe, which persisted for 6 months. Thus, he was referred to our hospital for bronchoscopy.

**FIGURE 1:**
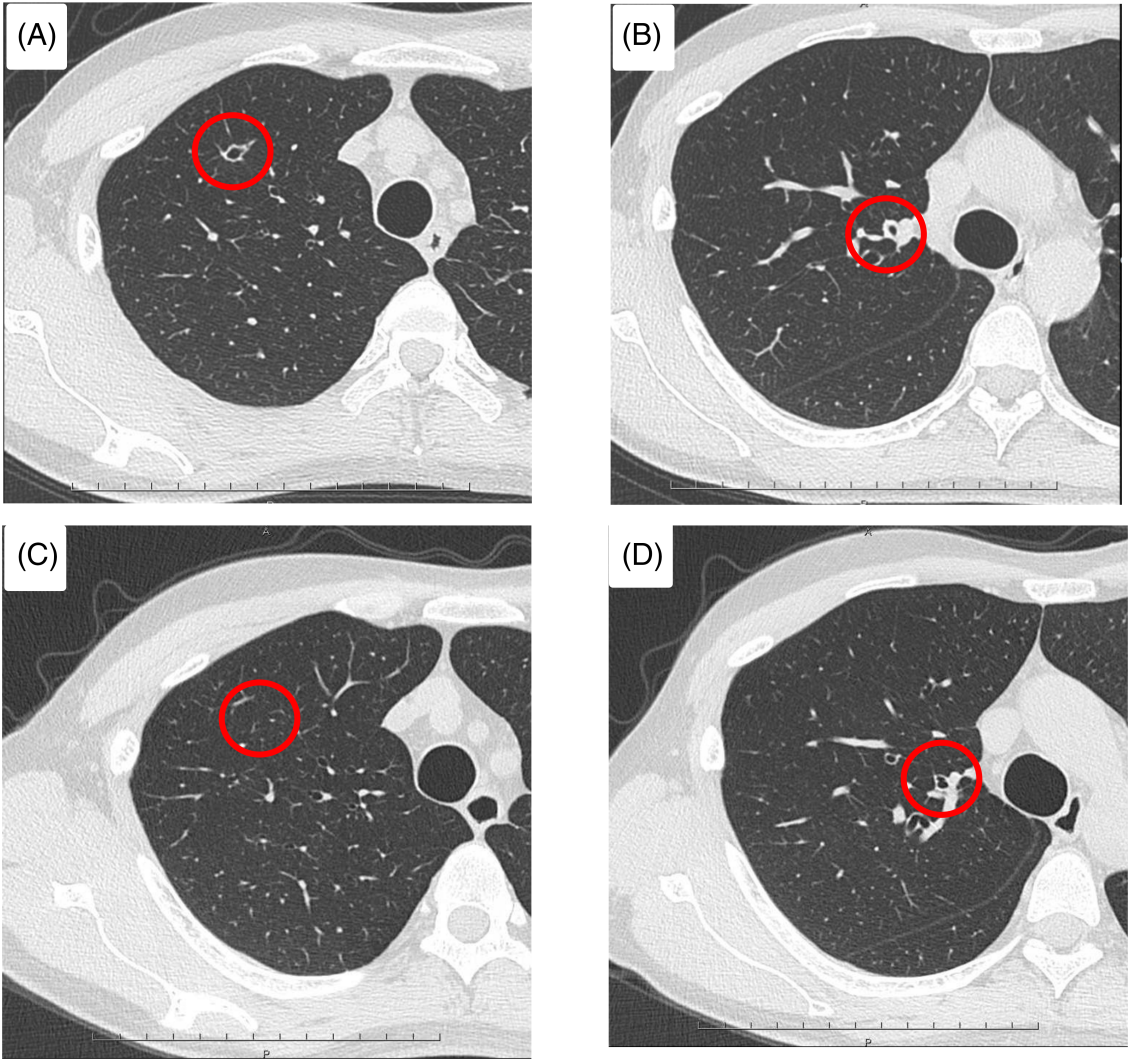
(A, B) High‐resolution computed tomography scan revealing focal bronchiectasis and bronchial wall thickening in the right upper lobe. (C, D) Bronchial wall thickening improved following treatment with fluconazole.

The patient did not have remarkable medical history. He was a farmer until the age of 34 years, being in close contact with wild birds and livestock. He did not have a smoking history. On examination, his temperature was 36.2°C, pulse rate was 97 bpm, blood pressure was 114/75 mmHg, respiratory rate was 16 /min, and oxygen saturation was 98% in ambient air. Auscultation revealed normal breathing sounds. Laboratory analyses yielded negative results for serum *Aspergillus* antigen and antibodies, serum cryptococcal antigen, human immunodeficiency virus serology, interferon‐gamma release, and serum anti‐glycopeptidolipid‐core immunoglobulin A titre. Chest high‐resolution CT (HRCT) revealed focal bronchiectasis and bronchial wall thickening around the entrance of the right B1 and B3 (Figure 1A, B).

**FIGURE 2:**
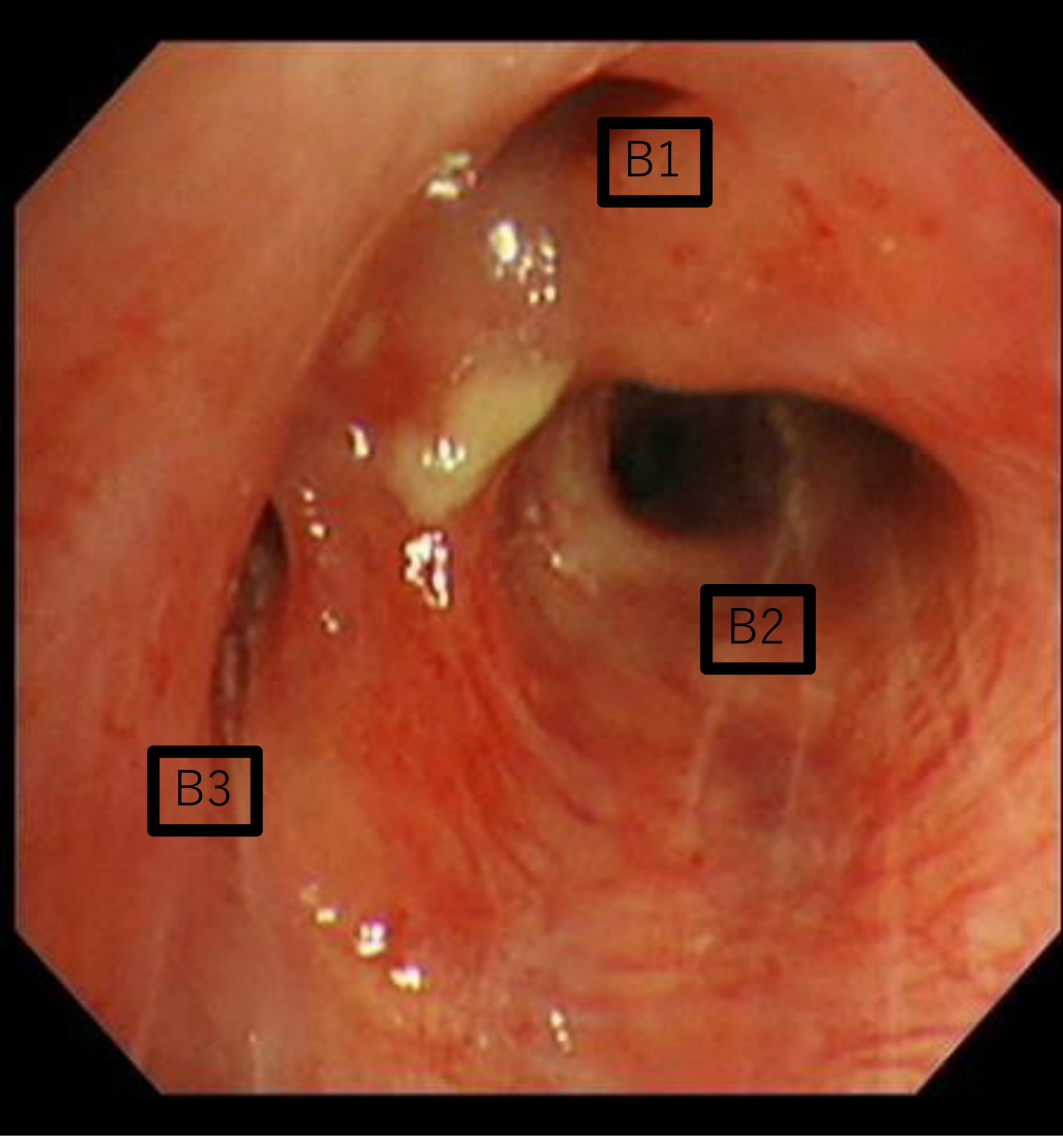
Bronchoscopy showing reddish and edematous mucosa, stenosed bronchi (right B1 and B3), and white moss at the bifurcation of the right upper bronchus. The B2 entrance area was normal.

Bronchoscopic findings revealed reddish and edematous mucosa, stenosed bronchi (right B1 and B3), and white moss at the bifurcation of the right upper bronchus (Figure 2). These findings were consistent with the areas of bronchial wall thickening observed on HRCT. There were no abnormal findings in the bronchial mucosa at other sites. Transbronchial biopsy and bronchial washing were performed at the bifurcation of the right upper lobe.

**FIGURE 3:**
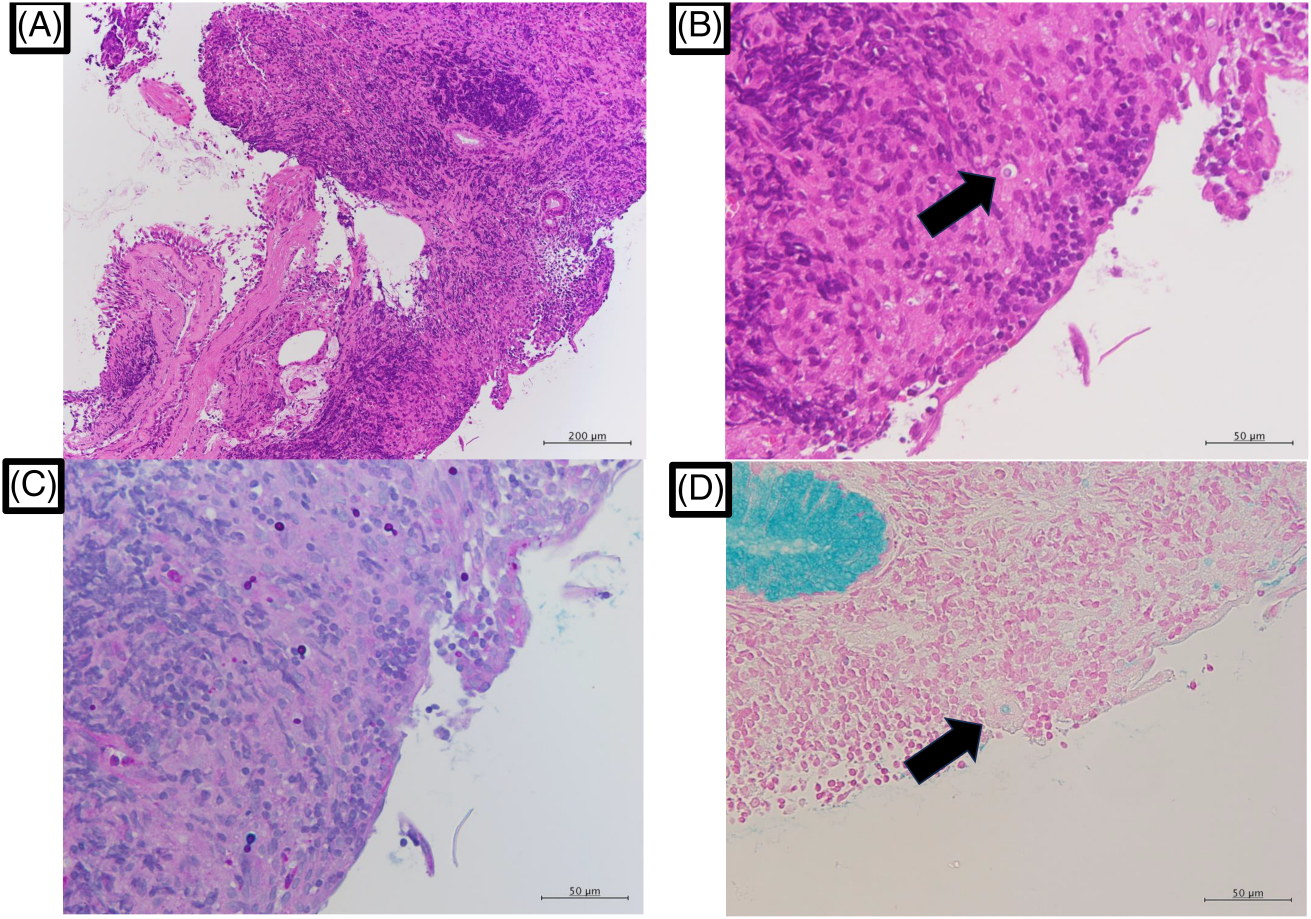
(A) Pathological image of bronchial mucosa captured during bronchoscopy. The examination revealed inflammatory cell infiltration and neovascular growth around the bronchioles. Staining by haematoxylin–eosin. (B) Circular structure with a halo sign present in the submucosa (arrow). (C) Alcian blue Periodic acid–Schiff‐stained image of the same pathological image as in (B) showing an encapsuled body (arrow). (D) Alcian blue‐stained image of the same pathological image as in (B) showing an encapsuled body (arrow).

Transbronchial biopsy showed inflammatory cell infiltration and neovascular growth around the bronchioles, which indicated bronchiolitis (Figure 3A). In addition, a round structure with a halo sign was recognized in the submucosa (Figure 3B), which was stained with Alcian blue Periodic acid–Schiff stain and Alcian blue stain (Figure 3C, D). These pathologic findings suggested endobronchial cryptococcosis. Bronchial washing for fungus culture revealed the presence of *C. neoformans*. Eventually, a diagnosis of endobronchial cryptococcosis due to *C. neoformans* was reached.

Examination of cerebrospinal fluid and brain magnetic resonance imaging with gadolinium enhancement confirmed the absence of meningitis. Thereafter, antifungal treatment with oral fluconazole at a dose of 200 mg/day was initiated. After 6 months of treatment with fluconazole, chest CT revealed improvement in the abnormalities (Figure 1C, D). After 6 months of treatment, bronchial wall thickening had improved, but remained present; therefore, treatment was performed for a total of 9 months. One month after treatment completion, the observed improvement was sustained. The patient has not experienced recurrence.

## DISCUSSION

This was a rare case of endobronchial cryptococcosis in a healthy adult without pulmonary parenchymal involvement. Although serum cryptococcal antigen analysis yielded negative results, bronchoscopic examination enabled us to reach a definitive diagnosis based on both pathological and microbiological findings.

*C. neoformans* is found worldwide without regional variation, and is often present in soil contaminated with excrement from pigeons and other birds. *C. gattii* is found on trees in tropical and subtropical regions. This fungus generally infects immunocompromised hosts, such as patients with human immunodeficiency virus/acquired immunodeficiency syndrome, and those receiving treatment with immunosuppressive drugs, such as corticosteroids. However, it may also occasionally cause lung lesions in healthy individuals who inhale the spores. Therefore, a thorough medical history taking is necessary. The patient reported close contact with wild birds 6 years earlier. The abnormality on CT was incidentally found during examination for the presence of COVID‐19 pneumonia at another hospital.

Cases of cryptococcal infections in patients with severe COVID‐19 who were treated with steroids have been previously reported.^2^ However, this patient had mild COVID‐19 without pneumonia, and did not require steroid treatment. Thus, an association between COVID‐19 and cryptococcal infection was considered unlikely.

Clinical manifestations of the disease include fever and chest pain. In immunocompromised patients, the lesions may spread to the central nervous system and cause meningitis, resulting in severe disorientation.

In previous cases, endobronchial cryptococcosis was often accompanied by infiltrative or nodular lesions in the lung parenchyma.^3^ To our knowledge, two cases of endobronchial cryptococcosis without lung field lesions were reported thus far. In both cases, the patients had poorly controlled bronchial asthma and received treatment with inhaled steroids.^4^, ^5^

Furthermore, serum cryptococcal antigen analysis is characterized by high sensitivity and specificity (i.e., >90%). All previously reported cases of endobronchial cryptococcosis with pulmonary lesions were positive for serum cryptococcal antigen,^3^, ^6^ whereas those with endobronchial lesions or endobronchial polyp lesions alone were negative for this antigen.^4^, ^7^ This evidence suggests that patients with endobronchial cryptococcosis alone may be negative for serum cryptococcal antigen.

When chest HRCT suggests subtle bronchial involvement in an immunocompetent host, endobronchial cryptococcosis cannot be excluded from the differential diagnosis even if serum cryptococcal antigen analysis yields negative results. In such cases, bronchoscopy should be performed to robustly confirm the cause.

## AUTHOR CONTRIBUTIONS

Susumu Sakamoto designed the study; Yui Shimanuki drafted the manuscript; Shion Miyoshi and Hiromichi Yoshida edited the manuscript; Noboru Iizuka critically reviewed the manuscript for important intellectual content; Satoshi Sonobe and Naobumi Tochigi discussed pathological views; Kazuma Kishi approved the submitted version of the manuscript. All authors read and critically revised all drafts of this manuscript. All authors read and approved the final version of the manuscript.

## CONFLICT OF INTEREST STATEMENT

None declared.

## ETHICS STATEMENT

Written informed consent was provided by the patient for the publication of this case report and accompanying images.

## Data Availability

The data that support the findings of this study are available from the corresponding author upon reasonable request.
